# Rapid quantification of clove (*Syzygium aromaticum*) and spearmint (*Mentha spicata*) essential oils encapsulated in a complex organic matrix using an ATR-FTIR spectroscopic method

**DOI:** 10.1371/journal.pone.0207401

**Published:** 2018-11-14

**Authors:** José Daniel Wicochea Rodríguez, Stéphane Peyron, Peggy Rigou, Pascale Chalier

**Affiliations:** UMR Ingénierie des Agro-polymères et Technologies Emergentes, Montpellier SupAgro, Université de Montpellier, INRA, CIRAD, Montpellier, France; University of New England, AUSTRALIA

## Abstract

Essential oils (EOs) are often encapsulated in various and complex matrices to protect them against potential degradation or to control their release. To achieve an optimum use in food products, their rapid and precise quantification after encapsulation and storage is required. Hence, a rapid ATR-FTIR method was developed and tested with two encapsulated essential oils (EOs): clove (*Syzygium aromaticum*) and spearmint (*Mentha spicata*);. Despite, the complexity of the matrix, this method coupled with univariate or multivariate regression models exhibited high potential for global quantification of the two encapsulated EOs. For clove EO, in relation to the major presence of eugenol and eugenol acetate, an analysis based on a unique band (1514 cm^-1^) was sufficient to obtain a good prediction with RMSEP value of 0.0173 g of EO per g of matrix. For spearmint oil which is characterized by numerous terpenoid compound, three bands (799, 885, and 1680–1676 cm^-1^) were suitable for a good prediction with RMSEP value of 0.0133. ATR-FTIR method was compared with a reference gas chromatography FID quantitative method in an EO release experiment and its efficiency was evaluated through modeling by the Avrami equation. Beside time saving, the ATR-FTIR method was also capable of monitoring the EO profile. This method could be easily adapted as a routine analysis in the EOs industry as quality control.

## Introduction

Essential oils (EOs) are a complex phytochemical mixtures of aroma compounds with a high value in a wide spectrum of industrial applications such as food and pharmaceutical ingredients but also fragrance or pesticide[1–6]. Regardless of the application, EOs are often used after encapsulation in synthetic or bio based materials to protect them against losses during processing and storage, to protect their components against heat-induced degradation, to stabilize them against unwanted changes, and to control their release or facilitate their manipulation[7–9]. Even after encapsulation, EOs can suffer alterations upon aging, and it is indispensable to monitor their composition during use and storage assuring quality[10,11]. Characterization and quantification of EOs are mostly based on precise chromatographic analytical techniques such as gas chromatography FID (flame ionization detector) and gas chromatography-mass spectrometry (GC-MS); this last would be the most used technique to identify and quantify unknown components in EOs. GC-MS is expensive and time consuming despite the extension of devices[12]. When pure essential oils are studied, only a dilution is needed using an adapted organic solvent. Nevertheless, when essentials oils are encapsulated or incorporated into a product, an extraction step is required before analysis, which is a time consuming procedure. In addition, the use of organic solvents needs to be limited in relation to their toxicity for humans and environment. However, GC-MS is a very sensitive method, which is powerful to the analysis of compounds present in traces with high accuracy and to early evidence the formation of new components responsible to off-flavor. Another mean to quantify and measure of the release of EOs encapsulated in complex matrices is the use of spectrophotometric techniques[13]. However, most of the aroma compounds present in EOs lack of a chromophore. Therefore, the global quantification of the EO could not be specifically studied by spectrophotometric techniques[14].

Consequently, there is a need to search alternative analytical methods that could include specific characteristics such as time efficiency, robustness, and sensitivity. Fourier transform infrared (FTIR) attenuated total reflectance (ATR) spectroscopy technique is rapid and requires minimal or no sample preparation; it provides a global fingerprint of a sample and can be considered as an alternative way for EOs quantification since signal intensity is proportional to concentration, but still a challenging procedure due to spectra superposition.

Pure EOs have been analyzed by infrared spectroscopic techniques to discriminate between different EOs for pharmaceutical and fragrance application,[15–17] to follow the release of EOs’ major components from polymeric matrices used for food packaging, [18–20] and to quantify EOs’ compounds directly on plant material.[21] Also, FTIR multivariate methods have been developed to quantify different plant metabolites other than EOs, such as sugars and organic acids[22–24] and to detect and quantify adulterations in butter and edible oils[25–27].

EOs exhibit a complex infrared spectral signature due to the high number of compounds including a wide variety of chemical groups. Given the fact that EOs share some same functional groups with biopolymers, the comparison of their spectra with encapsulation matrix could increase interference due to overlapping bands. This makes challenging the developing of a high quality prediction model to encapsulated EO.

To our knowledge, there are no previous studies that described the use of an ATR-FTIR method for the global quantification and monitoring the release of EOs trapped in a complex organic matrix. The aim of this study was to develop an ATR-FTIR spectroscopic quantitative methods of two specific EOs (clove and spearmint) encapsulated in the same complex organic matrix. The prediction models of the EOs quantification were developed using a univariate and a multivariate analysis regression. The obtained values were compared with a common GC-FID approach as external validation. The prediction model for the quantification of clove EO was applied in a release experiment where the amount of EO inside a complex matrix was monitored through time to study its robustness.

## Materials and methods

### Chemicals

Pure standard aroma compounds: (*R*)-carvone (≥98%), (*R*)-limonene (97%), eugenol (99%), *β*-caryophyllene (98.5%), methyl 2-hydroxybenzoate (≥99%), eugenol acetate (98%), 1,8-cineole (99%), and internal standard 2-heptanol (≥99%) were purchased from Sigma-Aldrich (St. Louis, MO, USA). Standards were used for GC calibration and FTIR identification. Clove essential oil from Madagascar and spearmint essential oil from India were purchased from Golgemma (Esperza, France). Hexane (>99% purity for analysis) used for dilution and solvent extraction of essential oil was obtained from Sigma-Aldrich. Ethanol (95% of purity) was used to clean the ATR crystal and was purchased from Groupe Meridise (France).

### Sample preparation of encapsulated essential oils

For encapsulation of essential oils, organic matrices used as carriers were formulated with a repeatable process that consisted in three formulation phases: 1) elaboration, 2) shaping and 3) drying. For confidentiality purposes the process cannot be detailed any further. The final matrices contained mainly starch, proteins, and a few amount of lipids. Raw matrices and matrices loaded with EOs were designed and stored at room temperature and controlled relative humidity (32%) until analysis.

### Preparation of EOs loaded matrices for standard calibration curves

For the ATR-FTIR standard calibrations, defined amounts of clove EO were carefully deposed inside a raw matrix using a precision micropipette, giving as a result a curve including 9 points corresponding to 0 mg and around 10.2 mg, 32.7 mg, 49.6 mg, 73.8 mg, 91.8 mg, 95.7 mg, 146.0 mg, and 192.7 mg of added EO per gram of matrix. In the same way, five amounts of spearmint EO were deposited inside of uncharged matrices; in this case the amounts of spearmint EO per g of matrix were around 8.7 mg, 50.1 mg, 98.9 mg, 148.6 mg, and 197.5 mg. For the GC-FID external validation, standard curves were elaborated using the same process of deposing EO inside of the raw matrix as described before. The amounts of clove EO added per gram of matrix were around 12.2 mg, 54.1 mg, 120.0 mg, 175.3 mg, and 251.5 mg and 14.2 mg, 49.3 mg, 94.2 mg, 152.9 mg, and 201.2 mg for spearmint EO. For both calibrations, each sample corresponding to one concentration (mg/g) was done in triplicate reporting the exact value of essential oil deposited by g of matrix, meaning that 27 concentrations were used for clove EO and 18 for spearmint EO taking into account the zero value.

### GC-MS and GC-FID analysis

Clove and spearmint EOs were characterized by a GC-MS (ISQ ThermoScientific, Austin, Texas, USA) equipped with a DB-WAX polar capillary column (30 m, 0.25 mm i.d. x 0.25 μm of thickness) and a quadrupole detector. Helium was used as carrier gas with a flow rate of 1.2 ml/min. The GC-MS oven temperature was kept at 40°C for 5 min and programed to 260°C at a rate of 2°C/min. One μL of diluted EO (1/100, v/v, in hexane) was injected at constant temperature of 250°C via an injector in split mode with a ratio of 1:20. Spectra were obtained in the electron impact mode with 70 eV of ionization energy, in full scan mode with a scan range between 40–500 amu. Linear retention indices (LRI) were obtained by simultaneous injection of samples and a series of alkanes (C7-C40, Sigma-Aldrich). The identification of components was based on the comparison of the determined LRI and those reported in the literature and mass spectra of libraries (NIST 2.0/Wiley/INRA). The quantification (expressed as a percentage) of each identified compound was done by comparing their peak area to the total area of the identified peaks.

EOs were extracted from the loaded or encapsulated matrices using hexane as solvent in the presence of 2-heptanol as internal standard (100 μL at 3g/L): ~0.3 g of matrix were stirred at constant rate (500 rpm) in a room temperature environment for 18h. The organic extracts were analyzed and quantified by a Varian 3800 GC (Les Ulis, France) equipped with a DB-WAX polar capillary column (30 m x 0.25 mm i.d. x 0.25 μm of thickness) and a flame ionization detector (FID; H_2_ 30 ml/min, Air 300 ml/min, Nitrogen 30 ml/min). Hydrogen was used as carrier gas with a flow rate of 1 mL/min. The oven temperature was kept at 40°C for 5 min and increased to 150°C at 2°C/min rate, then up to 250°C at 10°C/min rate with a detector temperature of 300°C. For quantification of major compounds, calibration curves were carried out by triplicate using 5 concentrations of each aroma compound in the presence of internal standard. The response coefficients were determined and used to achieve a precise quantification of major compounds.

### ATR-FTIR spectrum acquisition

The infrared spectra of clove and spearmint EOs, major EO compounds and raw matrices were characterized using a FTIR Nicolet Nexus 6700 spectrometer (Thermo Scientific, Courtaboeuf, France) in attenuated total reflection mode, equipped with a Mercury-Cadmium-Telluride High D detector and using the Omnic v7.3 software (Thermo electron). The spectra of major compounds of both EOs were obtained by deposing around 13 mg of the standard components over the diamond crystal (Ø = 2 mm) of a Smart DuraSamplIR accessory (Thermo Scientific, U.K.). Spectral data were collected at room temperature and accumulated from 64 scans with a resolution of 2 cm^-1^ in the range of 800 to 4000 cm^-1^. A correction was applied by subtracting the background spectrum of air. The matrices with a known amount of EO were grinded using a laboratory mortar until a fine powder was reached. Then, the powder was directly analyzed by the ATR-FTIR using the same procedure described above. The crystal was cleaned between measurements with ethanol and dried with a lint-free tissue. For each sample, three or four spectral data were performed. Then, for the calibration curve of clove EO produced from 9 concentrations by triplicate, 108 (N = 4 x 9 x 3) spectral data were acquired. For spearmint EO, 54 (N = 3 x 6 x 3) spectral data were taken.

### Chemometrics

Spectra were preprocessed using Omnic v7.3 software and a manual baseline correction by two points was applied to flatten the baseline. Spectral bands height were measured and calculated using the Omnic software. For unloaded matrices, the spectral variations were carried out on the spectra of the grinded matrices based on the 2900–2950 cm^-1^ spectroscopic band (wavenumber region used to normalize all spectra). Relative amount of clove EO was achieved applying univariate regression analysis using the TQ Analyst v7.3 software (Thermo electron). Regression was performed basing on the specific spectral band at 1514 cm^-1^ using 71 acquisitions (from the 108 spectral data) to construct the classical least squares (CLS) prediction model. The relative amounts of spearmint EO were achieved by multivariate regression analysis integrating three specific spectral bands at 885, 799 and 1676–1680 cm^-1^ using 36 acquisitions (from the 54 spectra data). Calibration models were validated by a cross validation (leave “one out” for each point of the standard curves) using a regression CLS calibration algorithm where the efficiency was measured with the coefficient of determination (*R*^*2*^) and the root mean square error of calibration (RMSEC) values. The models were then tested on an independent validation data set (*n* = 37 for clove EO [N = 108] and *n* = 18 for spearmint EO [N = 54]) where the performance is reported as the coefficient of determination of validation (*r*^*2*^*)*, the root mean square error of cross validation (RMSECV), and root mean square error of prediction (RMSEP). The standard error of prediction to the standard deviation of the reference data (RPD) was calculated. RPD values over a value of 5 could be considered as a good indicator for prediction purposes on quality control (Williams & Norris, 2001). The following equations were used:

$$RMSE\;(CV\;or\;P)=\sqrt{\frac{\sum_{i=1}^{n}{\left(y_{i}-{\hat{y}}_{i}\right)}^{2}}{n}}$$ (1)

$$Sd=\sqrt{\frac{1}{n-1}\sum_{i=1}^{n}{\left(y_{i}-\bar{y}\right)}^{2}}$$ (2)

$$RPD=\frac{Sd}{SEP}$$ (3)

Where *Y*_*i*_ is the actual value of EO deposed in the matrix, *ŷ*_*i*_ is the value of EO predicted by the model of the ATR-FTIR spectrum for the sample, *n* is the number of samples used in each data set, and *Sd* is the standard deviation for the EO quantity in the calibration data set. The limit of detection (LOD) and the limit of quantification (LOQ) of the CLS methods were calculated with the guidelines of the International Conference on Harmonisation (ICH)[28].

### Controlled release of clove EO from matrices

The clove EO controlled release experiment was carried out by introducing matrices loaded with a defined amount of clove EO inside a Memmert oven HPP IPP plus (Buchenbach, Germany) at controlled temperature of 25°C and relative humidity of 72% during a period of 34 days. At determined interval times, samples were taken out and the remaining quantities of clove EO were analyzed by the ATR-FTIR quantitative method and GC-FID conventional method for comparison purposes.

## Results and discussion

### GC-MS EOs characterization

The identification and characterization by GC-MS of clove and spearmint essential oils showed 21 and 25 different chemical compounds respectively (Tables 1 and 2). For clove EO, the two major compounds were two phenylpropanoids: eugenol and eugenol acetate, which constituted around 86% of the identified components. Clove EO exhibited also sesquiterpenes, which represented the second major class of components with *β*-caryophyllene (9.77%), *α*-caryophyllene or *α*-humulene (1.39%) and caryophyllene oxide. The other identified compounds including esters, alcohols, ketones, aldehydes, and one monoterpene compound were present at a very weak percentage <1%. Madagascar is the origin of the clove EO used in this study, but it showed a similar chemical composition as a Turkish clove bud EO where eugenol (87%) and eugenol acetate (8%) represented 95% of the whole EO at the detriment of *β*-caryophyllene (3.56%).[29] In contrast, the clove EO differed in composition and proportion of those identified by Hossain *et al*, (2014) and obtained from varieties of clove collected in different countries of the gulf regions. Indeed, the second major compound was *β*-caryophyllene (between 23 and 31% of the total EO) and the third eugenol acetate (~ 4 to 6%). Certainly, EOs composition can vary due to environmental and geographic conditions, agricultural practices, age and part of the plant used for extraction (*e*.*g*. leaves, flowers, roots *et cetera*), and the extraction process[1,4,6,21].

**Table 1:** Aroma compounds identified in clove essential oil and relative %.

Organic family	Compound	LRI	%
Phenylpropanoids	Eugenol	2158	62.52
	eugenol acetate	2253	23.62
	4-allylphenol	2430	0.47
Monoterpenoids	(*Z*)-*β-*ocimene	1238	0.02
Sesquiterpenoids	*α*-copaene	1424	0.12
	*β-*caryophyllene	1577	9.77
	*α-*caryophyllene	1646	1.39
	*α-*farnesene	1708	0.09
	caryophyllene oxide	1952	0.98
Alcohols	2-hexanol	1217	0.03
	linalool	1513	0.05
	*α-*cadinol	2201	0.04
	τ-cadinol	2003	0.09
Esters	2-heptyl acetate	1249	0.03
	benzyl acetate	1703	0.03
	methyl 2-hydroxybenzoate	1757	0.91
Ketones	3-hexanone	879	0.02
	2-nonanone	1340	0.02
	2-heptanone	1146	0.02
Aldehydes	3-furaldehyde	1421	0.05
	5-methyl-furfural	1517	0.01

**Table 2:** Aroma compounds identified in spearmint essential oil and relative %.

Organic family	Compound	LRI	%
Monoterpenoids	*β*-pinene	1012	1.77
	1-(*R*)*-α-*pinene	1097	1.67
	*β*-phellandrene	1113	0.95
	*β*-myrcene	1162	2.99
	(*R*)-limonene	1195	25.56
	*α-*Terpinolene	1169	0.31
	1,8-cineole	1199	3.04
	γ-terpinene	1237	0.46
	1-methyl-4-propan-2-ylbenzene	1262	0.51
	*p*-menthone	1446	0.5
	(*E*)-β-terpineol	1461	0.44
	(*Z*)dihydrocarvone	1591	1.62
	1,4-terpineol	1593	0.9
	(*R*)-carvone	1722	49.36
	(*E*)-carveol	1829	0.69
	(*Z*)-carveol	1859	0.3
Sesquiterpenoids	*β*-bourbonene	1501	1.93
	*α*-caryophyllene	1576	1.65
	(*Z*)*-β-*farnesene	1670	0.34
	(*R*)-germacrene	1691	0.35
Alcohols	3-octanol	1397	0.65
	4-ethylguaiacol	1607	0.32
	menthol	1635	1.98
Ester	menthol acetate	1554	0.60
	Unidentified compound	1032	0.17

Terpenoids were the predominant organic family for spearmint EO, including linear and cyclic components, oxygenated derived and esters. The two major compounds representing 75% of total oil were (*R*)-carvone (a terpene ketone) and (*R*)-limonene (a cyclic terpene). They are followed in less proportion by a terpene ether 1,8-cineole and a terpene alcohol, menthol, other monoterpenes such as *β*-myrcene, 1-(*S*) and 1-(*R*)-pinene, but also by sesquiterpenes such as *β*-bourbonene and *β*-caryophyllene. One derived compound of *R*-carvone, *(Z)-*dihydrocarvone was also found at a level >1%. On the basis of the biosynthetic pathway of *Mentha* species triggered by geographical conditions, they could be divided into carvone rich, menthol rich and pulegone/piperitone rich EOs[30–32]. The second reported major compound in the *M*. *spicata* EO is usually 1,8-cineole[30–32]. In this study, the spearmint EO used comes from India and is an EO rich in carvone with limonene as second major compound and not 1,8-cineole. The two major compounds of *M*. *spicata* EO were comparable with those reported for two *M*. *Spicata* species originated from India and Asian regions. [6,33] Comparing the composition of the two EOs, the only major compound found in both EOs was a bicyclic sesquiterpene (*β*-caryophyllene) with a difference of around 8% between the two EOs.

### Analysis and selection of the spectroscopic bands

The study of the matrices without added EOs revealed their high complexity. The matrices were composed of polysaccharides (starch and cellulose), lipids, and proteins. The matrix showed a main band corresponding to starch[34] which was centered at 1017 cm^-1^ with shoulders at 930 and 1078, and 1151 cm^-1^. Glucoside groups (C-OH) that correspond to starch fingerprint showed intensity at 1100–1000 cm^-1^, the increase of the band at 1000 cm^-1^ is also linked to water content in the starch matrix.[35] The rest of the signals corresponding to the matrix is mainly caused by C-H stretching vibrations modes between 2920 and 2851 cm^-1^ (symmetric CH_2_ and asymmetric CH_3_ and CH_2_) related to different compounds of biological material such as lipids, carbohydrates, proteins.[36,37] Stretching and bending modes at 1648 and 1542 cm^-1^ associated to protein content were also attributed to the matrix composition.[34,37]

**Fig 1:**
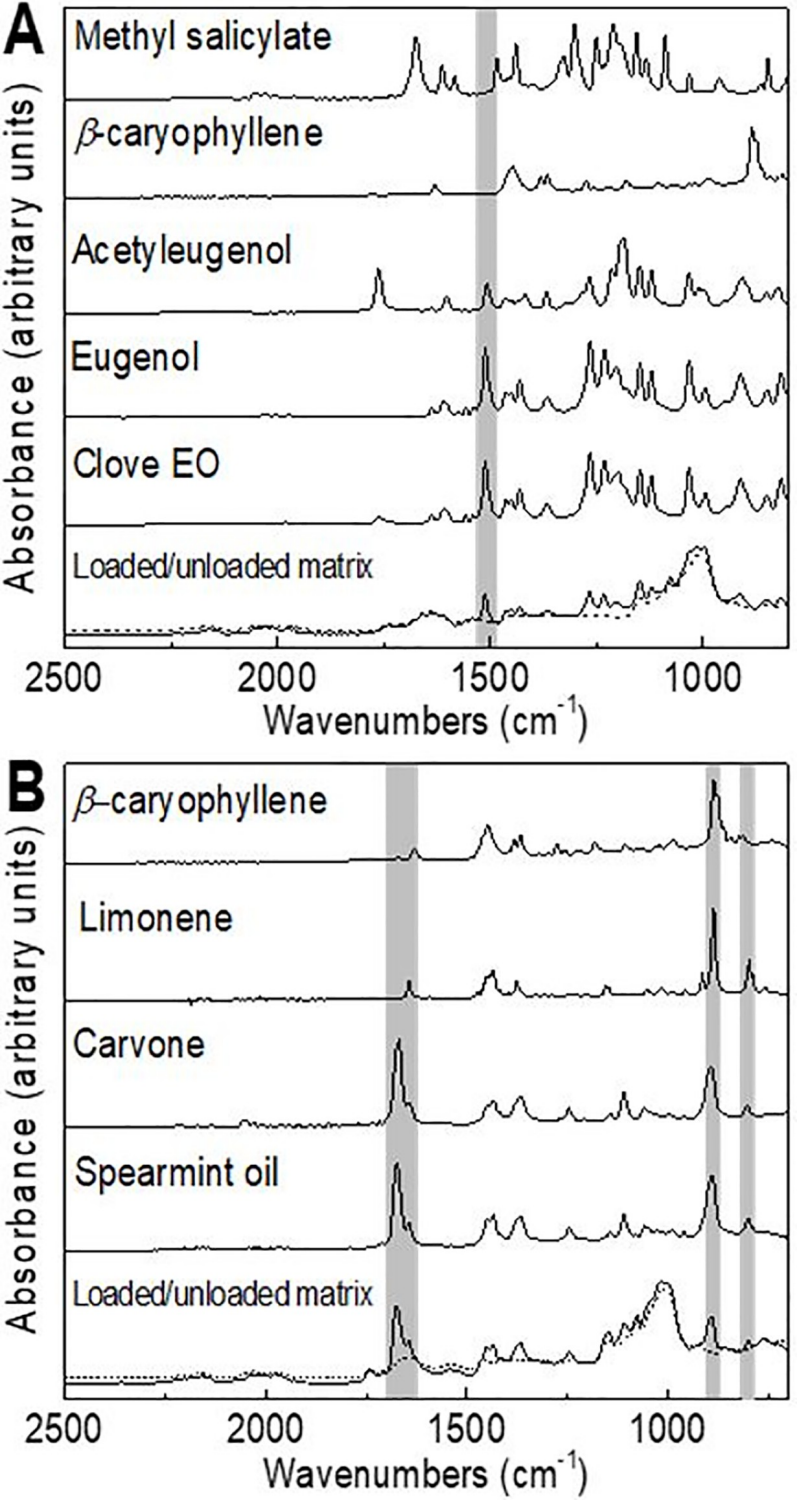
Major compounds of clove EO (A) and spearmint EO (B) with wave number regions for the CLS univariate and multivariate methods. Unloaded matrix represented with a dashed line.

When developing chemometrics quantification methods for EOs, most of them include the selection of a C = O stretching absorption band as it is broad and strong because of a large change in the dipole taking place in that mode.[21,27,38] This C = O bond is present in the functional groups of some terpenoids or phenylpropanoids as eugenol. Given the fact that in the current study the C = O spectral range (1705–1650 cm^-1^) overlapped the matrices fingerprint (interference problem); the spectra zones selected in the chemometrics analysis were chosen in function of the most prominent spectral bands corresponding to the EOs major compounds. It is important to notice that biological and functional activities of the EOs are mainly attributed to these compounds. Therefore, to identify and include these specific bands is primordial to assure the performance of the model. The spectra ranges highlighted in Fig 1 indicate the wavenumbers zones selected to perform the CLS multivariate analysis. The selected zones had a minimal superposition with the reference matrix spectral ranges which makes easier the identification and quantification of the EO.

First, a preliminary analysis was performed for clove EO by including the region range between 1000–1600 cm^-1^ to take into account specific vibration modes of its compounds. In this range, the stretching vibration modes of the aromatic C = C bond[18,39] (1514 cm^-1^) present in eugenol, eugenol acetate, and methyl 2-hydroxybenzoate and the asymmetric stretch C-O-C linkage of the ether functional group (1100–1210 cm^-1^)[37,40] present in eugenol were considered. However, due to poor quality results (data not shown) in the calibration/quantification model the zone range was reduced to a specific spectral band as highlighted in Fig 1A.The chosen C = C aromatic bond is present in the spectra of 4-allylphenol too. However, regarding its structure, the specific stretching vibration absorption bands of the allyl group are located at 1637 and 995 cm^-1^ [41,42]. For this reason, this compound has not been included in the quantification method. Stretching vibrations of C = C bond is known to be miscellaneous[43] since it is present in acyclic terpenes such as linalool and ocimene, and cyclic compounds with an allyl group in their structure like *α* and *τ−*cadinol, and cadinene. However, it is obvious that the band intensity is low or missing in the spectra of limonene (Fig 1B) an acyclic terpene. Therefore, it is necessary to analyze reference standards for all the compounds desired to identify or quantify to avoid miscalculations. In the same way the spectrum of *β*-caryophyllene did not showed this specific band as observed in Fig 1A. This implied that *β*-caryophyllene, *α*-caryophyllene and caryophyllene oxide could not be identified and quantified by considering only this specific band. Then, by selecting this band, the two major components eugenol, eugenol acetate of clove EO but also methyl 2-hydroxybenzoate were represented covering ~86.5 percentage of the total EO composition. The rest of compounds present in clove EO involved sesquiterpenes (12.3%), alcohols (0.24%), esters (0.97%), ketones (0.09%), aldehydes (0.06%) and a monoterpene (0.02%). One way to increase the sensitivity of the method would be to integrate the sesquiterpenes (12.3%) proportion into the model; however, this class lacks of a characteristic spectral band that could represent the global amount of this group. Concerning the spectrum of *β*-caryophyllene and its sesquiterpene nature, potential spectral regions (from 1446 to 800 cm^-1^) could be selected, but that implies spectra superposition with the unloaded matrix decreasing the confidence of specific compound identification. The addition of specific spectral regions characterizing the functional groups of the other EO compounds would include ketones (1750–1705 cm^-1^) alcohols (3600–3500 cm^-1^), and aldehydes (1740–1720 cm^-1^). However, as these compounds were in minority and their spectra overlapped with the unloaded matrix spectrum, we considered negligible the addition of these zones into a multivariate model.

For spearmint EO, monoterpenes represented the major proportion of the EO (91.1%) and among them, (*R*)-carvone is the major compound (49.4%). In consequence, the selection of a spectral band that corresponds to carvone is primordial. (*R*)-carvone exhibits a strong peak at the range of 1680–1676 cm^-1^ that corresponds to the stretching of its unsaturated carbonyl group[20,44]. The selection of this band involved the overlapping of peaks of other compounds present in spearmint EO. As an example Fig 1B shows the overlapping of a peak corresponding to limonene at 1643 cm^-1^. Spectra superposition can decrease the sensitivity of the chemometrics method if based on one compound. However, this was compensated by the addition of two strong appearing bands at 885 cm^-1^ and 799 cm^-1^ that correspond to the = C-H vibration of the disubstituted and trisubstituded double bonds respectively of (*R*)-limonene (25.56% of spearmint EO)[45,46]. The addition of these two spectral regions enabled a quantitative analysis of IR spectra despite the overlap of *β*-caryophyllene and (*R*)-carvone spectral bands. Table 3 summarizes the main IR absorption bands corresponding to the specific compounds of both essential oils used to the development of the proposed ATR-FTIR method. In short, for clove EO, only one band could be used to build a model while three bands will be needed for Spearmint EO.

**Table 3:** Main IR absorption bands used as reference for the elaboration of the ATR-FTIR method.

Wavenumber (cm^-1^) and vibration mode	Chemical functional group	Compound
1514 stretching	Aromatic C = C	Eugenol and eugenol acetate
1680; 1676 stretching	Carbonyl C = O	Carvone
885 bending	Disubstituted C-H	Limonene
799 bending	Trisubstituted C-H	Limonene

### Impact of matrix aging on the spectral fingerprint

**Fig 2:**
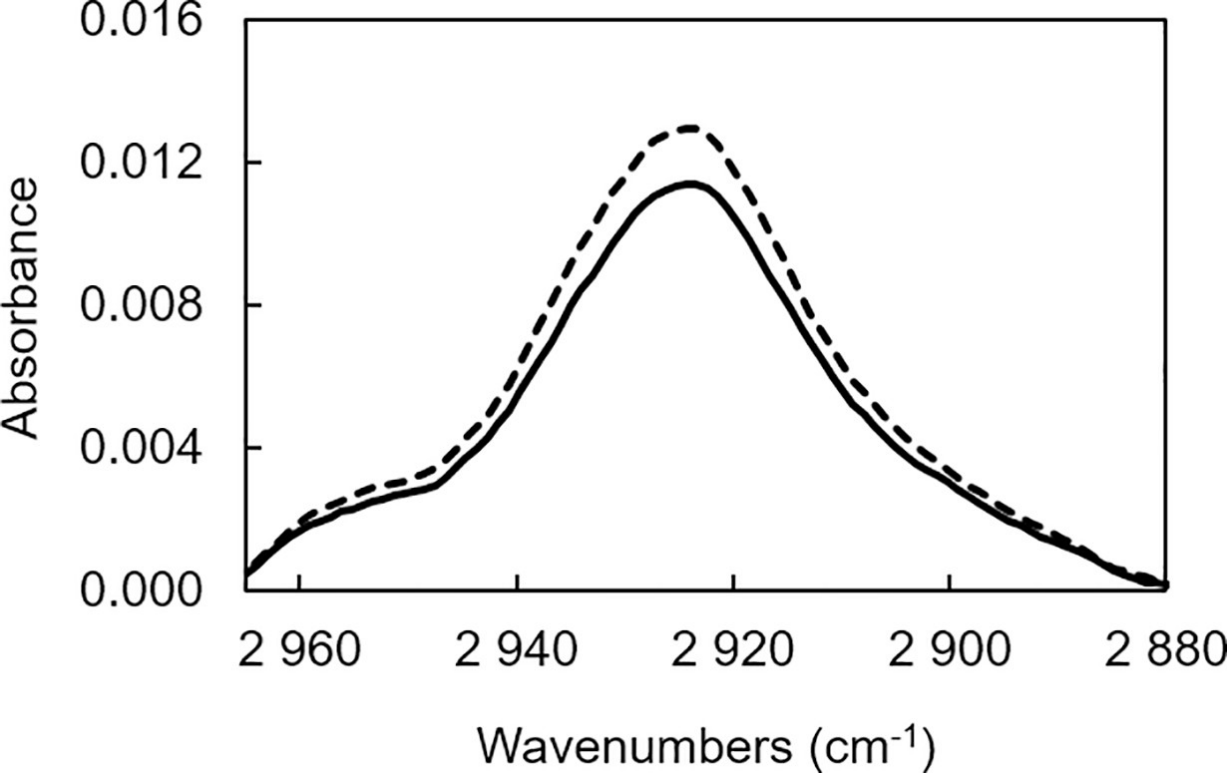
Evolution of the normalization spectral band (2900–2950 cm^-1^). Solid line represents the matrix after the elaboration process (*n =* 15) and the dotted line the matrix after three months (*n* = 15).

The investigation of EO release kinetics from the organic matrix required to preliminary ensures that the spectral fingerprint of the matrix is invariant over time. This all the more important because the quantitative analysis of EOs is performed on specific bands that are measured on standard spectra normalized using a specific band of the matrix. In this way and considering the specific vibration frequency of the material, the normalization was performed on the C-H stretching band of aliphatic methylene group between 2920 and 2851 cm^-1^. The evolution of this band which was considered as an indicator of the material aging was investigated through the spectral subtraction established on the mean spectra acquired on initial material and after three months of storage at room temperature (Fig 2). The linear response issued from this treatment demonstrated the stability of the signal in this range of frequencies and provided evidence of the use of this band for the normalization procedure of the spectra for the quantitative analysis of EO.

### Quantitative analysis of encapsulated EOs by ATR-FTIR

**Fig 3:**
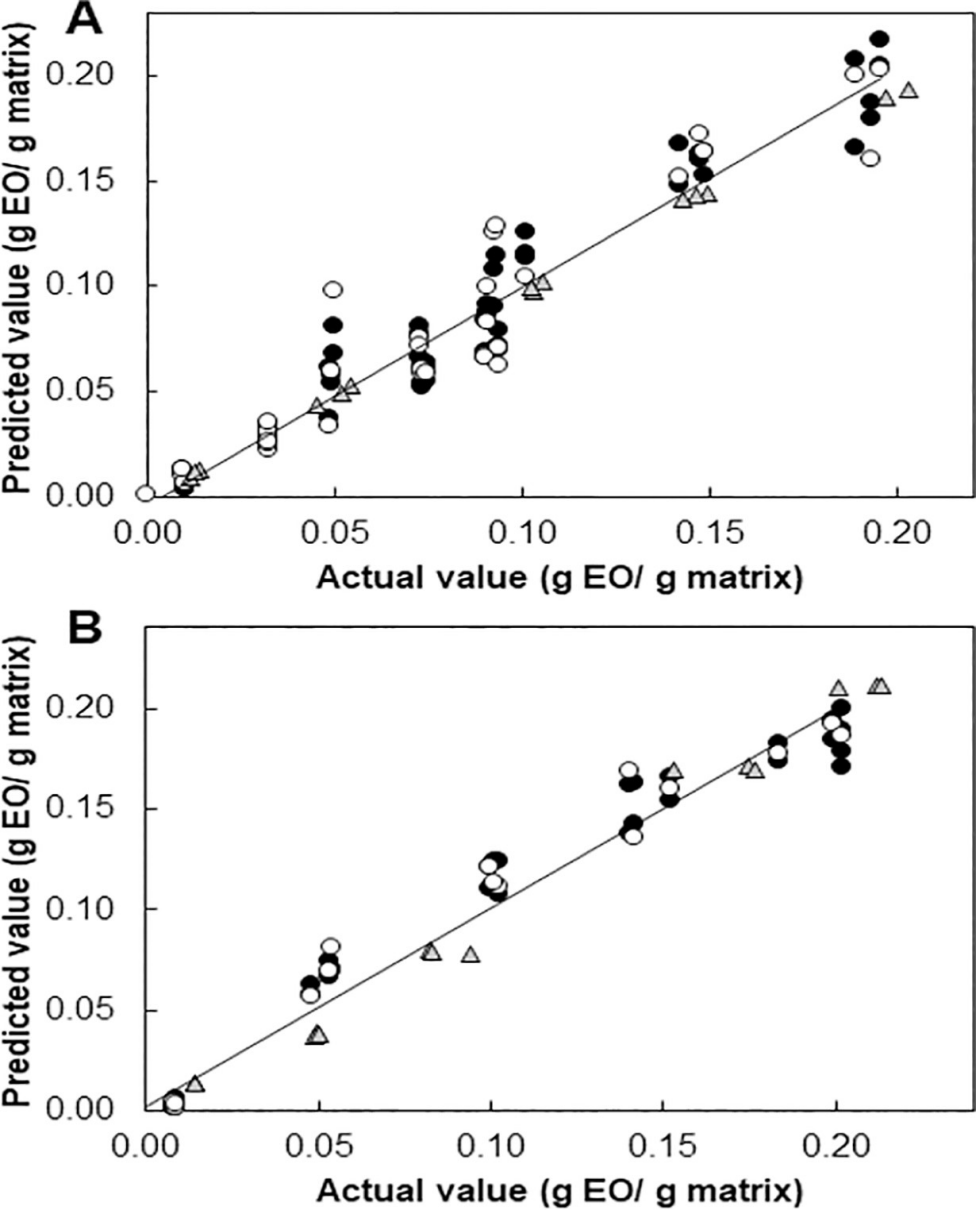
Results of the calibration and cross-validation CLS quantification method and GC-FID reference method. Clove EO (A) and spearmint EO (B). ATR-FTIR calibration values (●), ATR-FTIR validation values (○), and GC-FID quantification values (Δ).

Fig 3 shows the results for GC-FID quantification of EOs as well as the resulting ATR-FTIR based calibration and validation models using the pre-selected bands for each EO. For clove EO, spectroscopic data was composed of nine concentrations and each one of them was performed by triplicate. For each sample, four collected spectra were acquired by deposing around 13 mg of sample (grinded matrix enriched with EO) over the ATR crystal. From the total of 108 scans obtained, 71 were selected to construct the prediction model and 37 spectra acquisition were taken out to build the validation model. For spearmint EO calibration data consisted in three collected spectra per sample corresponding to one concentration. Triplicates were performed for each one of them, resulting in a curve of six concentrations. From the total of 54 acquisition obtained, 36 were selected to construct the prediction model and 18 scans were taken out to build the validation model. In these conditions, for both EOs, accurate predictions were obtained as indicated by the high values of *R*^2^ (>0.95) (Table 4). Low values of RMSEC, RMSECV and RMSEP were aimed to achieve the best performance of the method. RMSEC expressed the average calibration error of the models, RMSECV represents the errors calculated on the method giving an estimate of how the model is built on the data set to predict values for unknown samples and last, RMSEP is an indicator of the performance of the model by stating the prediction error. These values are also reported in Table 4. Lower values of RMSEP were observed in the spearmint EO quantification model than the ones found in the clove EO quantification model. This demonstrated that spearmint EO quantification model is more reliable than the one used for clove EO. Moreover, bias is negligible (Table 4), indicating no considerable systematic errors in the results given by the ATR-FTIR quantification method. RPD calculated value from all the data set of clove EO is higher than 6, indicating a good ability for quantification. In the other hand, the RPD calculated value for spearmint EO was higher, which means that the application for this method could go from screening to quality control[47]. This illustrates that the selection of spectral regions which represent the major aroma compounds of EO and having the minimum superposition with the matrices spectra provided a good global quantification. As suggested by Gudi *et al*., (2015)[21] and Krähmer *et al*.,(2013)[38] the increase of samples and repetitions allows to improve performance and robustness of the calibration. It is the reason why in this study more repetitions were done for clove EO compared to spearmint EO.

**Table 4:** Statistical data of ATR-FTIR CLS model for encapsulated clove and spearmint EOs.

EO	*r*^2^	R^2^	RMSEP	RMSEC	RMSECV	Bias	LOD	LOQ	RPD
Clove	0.9142	0.9551	0.0173	0.0133	0.0172	3.38x10^-5^	0.0032	0.0107	6.64
Spearmint	0.9677	0.9689	0.0133	0.0131	0.0132	1.53x10^-4^	0.0039	0.0132	8.00

Regarding the sensitivity of the ATR-FTIR method, acceptable LODs were obtained with values inferior to 0.004 g EO/g matrix (Table 4). These values were obtained by multiplying by 3 the standard deviation measured on 15 blank samples and dividing the resulting value by the slope of the ATR-FTIR standard curve. To calculate the LOQs values, same procedure was followed, but standard deviation values were multiplied by 10 instead of 3. These values are relatively high but are adapted to the quantification of matrices enriched in EOs. Last statements are supported by the results obtained with the standard extraction followed by quantification GC procedure. Indeed, the lowest concentration point of the calibration curve analyzed by ATR-FTIR reached 0.0139±0.0002 g of spearmint EO corresponding to 0.0142±0.0012 g of EO deposed for 1g of matrix. In the same way, matrices loaded with 0.0128±0.001 g of clove EO showed 0.011±0.002 g of EO by the GC-FID analysis. The comparison of the ATR-FTIR calibration curves with those obtained by GC-FID showed a good correlation. Bias between the determination coefficients of validation from both ATR-FTIR and GC-FID methods were 0.0057 for spearmint EO and 0.0222 for clove EO, considering as reference values, the GC-FID results. Results obtained between the ATR-FTIR and GC-FID calibration curves proved that it is possible to detect and quantify low amounts of clove and spearmint EOs by the ATR-FTIR method without time consuming for extraction.

### Controlled release and monitoring of clove EO

**Fig 4:**
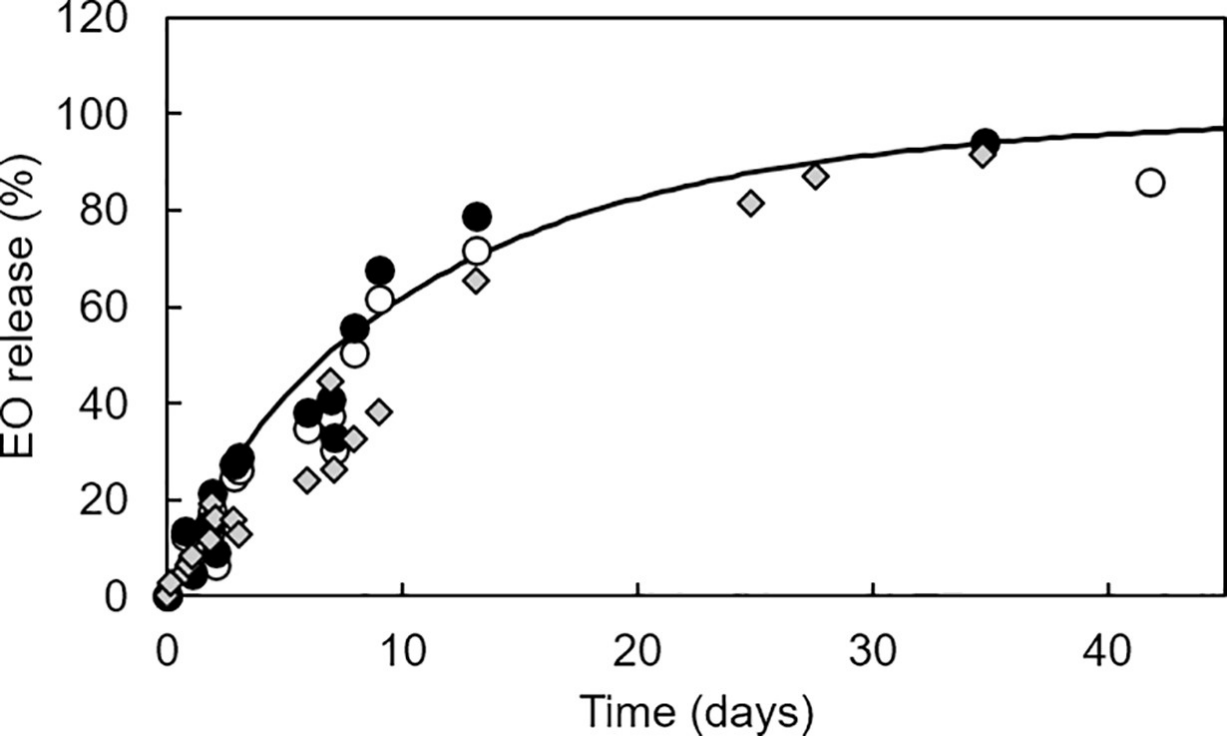
Clove EO release determined by ATR-FTIR (●) and GC-FID methods (○ = all components quantified, ♦ = eugenol, eugenol acetate, and methyl 2-hydroxybenzoate quantified).

As the amount of clove EO in the organic matrix was well quantified by the ATR-FTIR method, this method was used to follow the release of EO in controlled conditions. The results of clove EO release at controlled relative humidity (72%) and temperature (25°C) are reported in Fig 4. The released fraction was expressed by calculating the clove EO mass at a given time over the initial EO loaded mass and multiplied by 100. In parallel, the release was quantified by GC-FID method taking into account: (i) all the compounds present in the clove EO and (ii) only the three compounds characterized by the same spectral band (1514 cm^-1^) and quantified by the ATR-FTIR method. Averaged spectra for each matrix at given time submitted to the ATR-FTIR method gave values of remaining clove EO well correlated with the GC-FID method, regardless the compounds taken into account. This was expected because the targeted spectral band corresponded to the 86% of the total compounds present in the clove EO. At some points, relatively large variation (12.2–16.7%) could be observed possibly due to the different amounts of sample used in the two methods. Spectroscopic data were composed of five measurements of approximately 13 mg of grinded matrix. In comparison, for GC-FID the whole matrix extraction (approximately 0.3 g) was analyzed.

Furthermore, to identify the transfer behavior of the clove EO from the matrix, the equation of Avrami was applied to model the release experiments:

$$X=1-{e^{-kt}}^{n}$$ (4)

where *X = M*_*t*_*/M*_*0*_, *M*_*0*_ is the total amount of essential oil contained in the matrix at time 0 of the release experiment, *M*_*t*_ is the amount of essential oil retained in the matrix at a given time. *k* is the release constant (h^-n^), and *n* is a parameter used to represent the release mechanism. By taking a double logarithm of both sides of Eq (1) yields to Eq (2):

ln[−ln(1−X)]=nlnk+nlnt (5)

**Fig 5:**
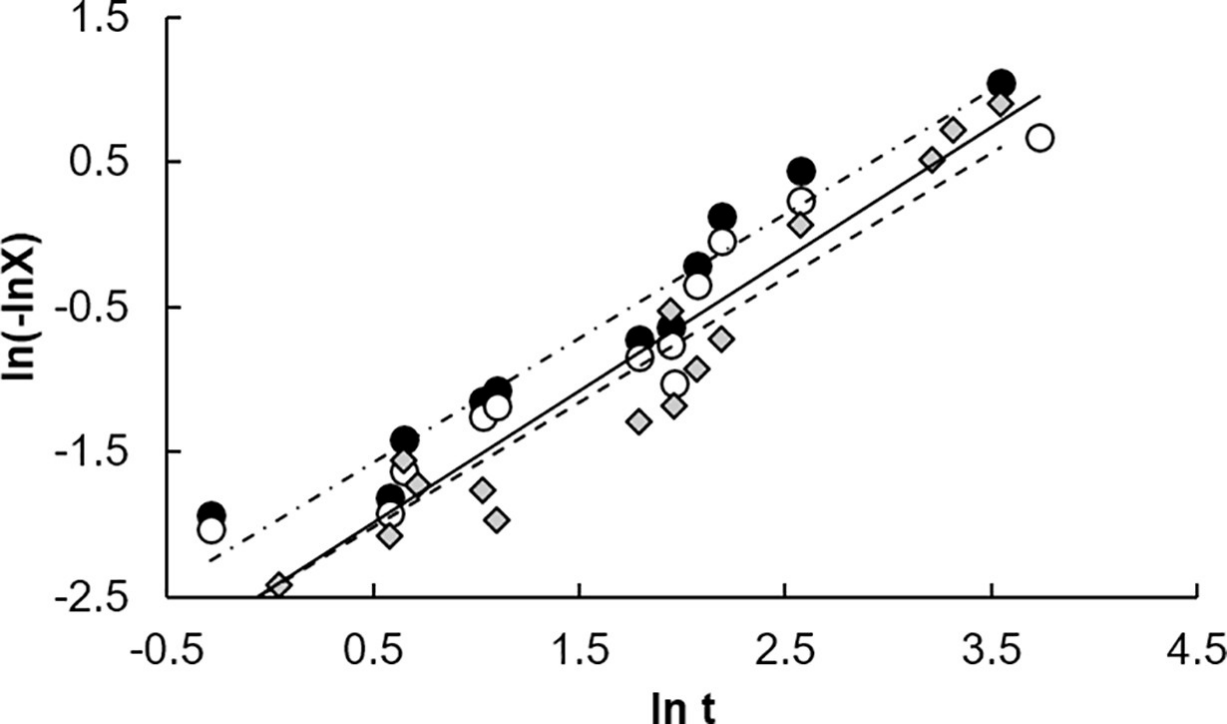
Correlation of release through time of clove EO by the equation of Avrami. ATR-FTIR quantification method values (●; dashed point line), GC-FID all components quantified (○; straight line), GC-FID eugenol, eugenol acetate, and methyl 2-hydroxybenzoate quantified (▲; dashed line).

From Eq (2) and by plotting ln(-ln *X*) against ln*t*, the release rate constant *k* and *n* values were calculated: the slope of the curve gives the *n* parameter and the release rate constant *k* from the interception at ln*t =* 0. Whatever the method used, the global release of clove EO presented a good correlation when plotting ln(-ln *X*) against ln*t* as shown in Fig 5. The statistical summary where the correlation coefficient values for the ATR-FTIR, GC-FID (all compounds), and GC-FID (eugenol, eugenol acetate, and methyl 2-hydroxybenzoate) are shown in Table 5. Given the increase of the variation or the total sum of squares value, it is obvious that increasing the number of compounds decreased the robustness of the model used to predict the release of clove EO.

**Table 5:** Statistical data of clove essential oil release experiment by ATR-FTIR and GC-FID method.

Release quantification method	Coefficient of determination (*R*^*2*^)	Transfer type (*n*)	Constant release (*k*)	Regression sum of squares	Total sum of squares
ATR-FTIR	0.95	0.86	0.14	8.44	13.83
GC-FID ^a^	0.84	0.91	0.09	12.64	33.12
GC-FID^b^	0.94	0.86	0.09	26.46	58.22

^a^ considering all the peaks

^b^ considering only eugenol, eugenol acetate and methyl 2-hydroxybenzoate

Relying on the *n* value, the transfer nature can be defined. The obtained *n* values (Table 5) suggested that the transfer of the EO was an anomalous transport phenomenon and was not a Fickian type[48]. Anomalous transfer mechanism could be due to a relaxation of the matrix[49]. The similar values of *n* found for GC-FID and ATR-FTIR considering the same compounds proved that the characterization of the release phenomena could be obtained by the adapted ATR-FTIR model. However the slight difference of *k* values between the two methods could be explained by the aromatic profile evolution. Indeed, during the release of the clove EO, it was observed by the GC-FID method that the relative proportion of eugenol and eugenol acetate changed with a higher content of the first compared to low the second. This change could be explained by the slow release of eugenol and the fast release of eugenol acetate. However, the two compounds have similar levels of vapor pressure at 25°C and the ester is characterized by higher molecular size suggesting more slow release than eugenol. Nevertheless, the initial content of eugenol did not match the initial proportions of the EO referenced by the GC-MS analysis suggesting changes in aromatic profile after encapsulation process. Hence, it is possible that hydrolysis of the ester function of eugenol acetate occurs due to high moisture content and temperature during the encapsulation process. Then, the ester is turning into eugenol. This explains why a higher proportion of eugenol was quantified by the GC-FID method. In contrast, the change of the aromatic profile did not affect the quantification by ATR-FTIR method. Besides, a strong spectral band at 1760 cm^-1^ region is found the eugenol acetate and clove EO reference spectra (Fig 1A). This peak could correspond to the ester stretching carbonyl group of eugenol acetate. During the release experiment, the peak at 1760 cm^-1^ was observed in the spectra in agreement with eugenol acetate presence. However, because the intensity of the band was low and eugenol acetate was already taken into account in the specific 1514 cm^-1^ spectral region, the spectrum zone at 1760 cm^-1^ was not included in the regression analysis. Nevertheless, if specific quantification is required, the method could be rebuilt by adding the corresponding spectral region of eugenol acetate and quantified it separately. Thus, the ATR-FTIR method could additionally be used to monitor profile change due to degradation reactions of the EOs during processing or storage.

## Conclusions

This study investigated the use of ATR-FTIR technique for determining the global content of clove and spearmint EOs trapped in a starch and proteins based matrix. The developed ATR-FTIR prediction models were based on univariate or multivariate regression analysis after selection of spectral bands. Only identified characteristic spectral bands of major compounds were included in the models because they strongly govern the variability between the matrices with different EO loads. For clove essential oil a univariate analysis regression is successfully applied while for spearmint oil multivariate analysis regression based on 3 bands is needed for good prediction. Although the plurality of aroma compounds of the EO composition and the presence of compounds at low concentration, this approach based on specific bands proved to be reliable, robust and appropriated for a global quantification of encapsulated EOs. For both EOs, the models were found of comparable quality than the GC-FID analysis reference method. In addition, the method shows the possibility to follow the global release of encapsulated EO without valuable loss of information and to monitor changes in the EOs profile, *e*.*g*. an ester hydrolysis. This is a critical point to follow the quality of food applications. The ATR-FTIR method can be adapted for a global quantification of EOs encapsulated in a complex matrix. By developing a specific calibration of each EO, this method can be used as routine analysis to follow the evolution of the aroma profile during storage. This technique allows a reduced time of analysis (minimal sample preparation) and has the advantage to be more chemical sustainable since there is no need of organic solvents for extraction.

## Supporting information

S1 Dataset Spectral data. Raw ATR-FTIR spectral data of samples and CLS.
(XLSX)
